# Simulated Impact of RTS,S/AS01 Vaccination Programs in the Context of Changing Malaria Transmission

**DOI:** 10.1371/journal.pone.0032587

**Published:** 2012-03-06

**Authors:** Alan Brooks, Olivier J. T. Briët, Diggory Hardy, Richard Steketee, Thomas A. Smith

**Affiliations:** 1 Department of Epidemiology and Public Health, Swiss Tropical and Public Health Institute, Basel, Switzerland; 2 University of Basel, Basel, Switzerland; 3 Malaria Control and Evaluation Partnership for Africa, PATH, Seattle, Washington, United States of America; Menzies School of Health Research, Australia

## Abstract

**Introduction:**

The RTS,S/AS01 pre-erythrocytic malaria vaccine is in phase III clinical trials. It is critical to anticipate where and how it should be implemented if trials are successful. Such planning may be complicated by changing levels of malaria transmission.

**Methods/results:**

Computer simulations were used to examine RTS,S/AS01 impact, using a vaccine profile based on phase II trial results, and assuming that protection decays only slowly. Settings were simulated in which baseline transmission (in the absence of vaccine) was fixed or varied between 2 and 20 infectious mosquito bites per person per annum (ibpa) over ten years. Four delivery strategies were studied: routine infant immunization (EPI), EPI plus infant catch-up, EPI plus school-based campaigns, and EPI plus mass campaigns. Impacts in changing transmission settings were similar to those in fixed settings. Assuming a persistent effect of vaccination, at 2 ibpa, the vaccine averted approximately 5–7 deaths per 1000 doses of vaccine when delivered via mass campaigns, but the benefit was less at higher transmission levels. EPI, catch-up and school-based strategies averted 2–3 deaths per 1000 doses in settings with 2 ibpa. In settings where transmission was decreasing or increasing, EPI, catch-up and school-based strategies averted approximately 3–4 deaths per 1000 doses.

**Discussion:**

Where transmission is changing, it appears to be sufficient to consider simulations of pre-erythrocytic vaccine impact at a range of initial transmission levels. At 2 ibpa, mass campaigns averted the most deaths and reduced transmission, but this requires further study. If delivered via EPI, RTS,S/AS01 could avert approximately 6–11 deaths per 1000 vaccinees in all examined settings, similar to estimates for pneumococcal conjugate vaccine in African infants. These results support RTS,S/AS01 implementation via EPI, for example alongside vector control interventions, providing that the phase III trials provide support for our assumptions about efficacy.

## Introduction

RTS,S/AS01 (RTS,S) is the most advanced malaria vaccine under development. Work started in the 1980's, and the phase III, or final trial prior to seeking regulatory approval, began in 2009 in Africa [1]. Interim phase III results suggest efficacy against first or only clinical episode of over 50% [2]. If all goes as planned, the trial could be completed around 2015.

At the same time, declines in malaria transmission have been documented in many areas of Africa [3]–[6]. While these changes have not been completely explained, and in some places transmission remains stable [7] or may even be increasing, the growing use of long lasting insecticide treated bednets (LLINs) and improved medicines for treating malaria are probably major causes of the declines [8]–[16]. However, malaria continues to kill close to 800,000 people each year, mostly among children under five years of age living in Sub Saharan Africa [6] and a malaria vaccine could be a valuable, additional tool to prevent malaria mortality and morbidity. It is to be hoped that we will see this toll further reduced by the time RTS,S is available, but both insecticide and drug resistance represent major threats [17], [18] and malaria programs remain dependent on financially-challenged donors [19]. It seems likely that RTS,S will be introduced into a complex mixture of changing patterns of transmission.

Two major mathematical models of malaria and pre-erythrocytic malaria vaccines, such as RTS,S, have been published in the past ten years [20], [21]. Both models run probabilistic micro-simulations in populations of thousands of people. The models are systematically fitted to an extensive library of field data from varying transmission settings. The transmission in an area is reflected by the entomological inoculation rate (EIR), which is a direct measure of the number of infectious mosquito bites per person per annum (ibpa). In the published model studies, the EIR is modeled at fixed levels of 2, 5, 11, 20, 42, 84, and 168 infectious bites per annum (ibpa) [22]–[24] or 3, 43, 46 81, 586, and 675 ibpa [20].

These studies projected that RTS,S is likely to have an important and varying public health impact, depending on level of transmission in an area and delivery strategy [20], [22]–[24]. Impact is quantified in terms of reduced transmission and/or cases and deaths averted. Impact on cases and deaths, particularly when delivered to infants via the Expanded Programme on Immunization (EPI), is thought likely to be greater in lower to moderate transmission settings. This is because, in partially protected individuals in higher transmission settings, vaccination is predicted to have an effect of shifting some of the burden of disease to older ages, rather than completely averting it [22], [23].

The decision to use RTS,S will partially depend on the impact sought. For many vaccine preventable diseases, a significant part of the impact is achieved by transmission reduction via herd immunity. For malaria transmission reduction, the initial transmission level and the feasibility of the delivery strategy are critical considerations. Modeling studies suggest that RTS,S will only have a substantive impact on transmission in areas where it is already low (e.g. 3 ibpa) [20], and when delivered through mass campaigns, reaching a large proportion of the entire population [20], [24]. At low EIRs, such as 2 ibpa and below, transmission becomes more focal and less stable.

EPI programs have delivered infant vaccines in developing countries for decades. Among African infants in 2009, 73% were immunized with three doses of Diphtheria-Tetanus-Pertussis (DTP3) vaccine scheduled at approximately 6, 10 and 14 weeks of age [25]. This is the same initial schedule as is being anticipated for RTS,S. The current clinical trials of RTS,S and regulatory plans include children aged six weeks through 17 months at first RTS,S vaccination. EPI and catch-up strategies, targeting this age range, thus represent the near-term options for implementing RTS,S. When delivered through EPI, RTS,S is likely to have greatest efficacy and impact in low to medium transmission settings [22], [23], and to be most cost-effective in areas with EIR from 2–20 ibpa [26]. This suggests that the value of RTS,S, as a complementary intervention to save lives and prevent morbidity, is likely to grow in importance with anticipated decreasing transmission trends resulting from the scaling up of malaria interventions across Africa. Impact through EPI is questionable in very high transmission settings (e.g. 168 ipba), where a large proportion of disease episodes prevented early in life may simply be experienced with a delay and are thus not fully prevented.

Vaccine delivery strategies other than EPI, such as school-based and population-wide mass campaigns, target incrementally wider age ranges and greater numbers of people. Mass campaigns are estimated to have relatively little impact on mortality and morbidity at a transmission above an EIR of 5 ibpa [23]. School-based and mass campaign strategies for delivering RTS,S may require additional multi-year clinical studies and regulatory decisions, beyond those currently anticipated. They may also require large, specialized initiatives for which there is limited precedent, suggesting feasibility studies would be needed.

The recognition that RTS,S may have varying efficacy and associated impact in different transmission settings has been considered in the design of its phase III trial: the trial includes 11 sites spread across Burkina Faso, Gabon, Ghana, Kenya, Malawi, Mozambique, and Tanzania, intended to reflect the wide range of transmission levels seen in Africa [1]. While possible to select sites over a range of transmission levels, it was not possible to select sites according to changing transmission dynamics.

RTS,S is, however, likely to be implemented in environments where baseline malaria transmission levels are changing. In some countries still scaling up interventions, transmission will likely be decreasing. Other countries, after initial successes, may be struggling to maintain suppressed malaria transmission levels and may see malaria transmission increase. Predictions of the potential impact of RTS,S, to be used by policy-makers at global and country levels [27], [28], will need to consider such contexts of changing transmission. It is unclear if the findings of models using fixed annual transmission levels hold where annual transmission is changing.

This paper uses simulations to examine how, depending on the delivery strategy, the expected impact of RTS,S may be affected when implemented in the context of increasing or decreasing malaria transmission trends.

## Methods

### Simulation model

The models are designed in a modular way to reflect the pathway from infection to development of disease and death as well as transmission via the vector and hence herd immunity effects. The base model [29], comprised modules for acquisition of *P. falciparum* infections [30], for regulation of parasite densities [31], for morbidity [32], for mortality [33] and for case management [34]. The drivers of uncertainty in this model were recently analyzed using a probabilistic sensitivity analysis [26]. The model ensemble used in the present study made use of 13 additional models with different assumptions about decay of immunity, heterogeneities in exposure, case management, susceptibility and comorbidity. Comprehensive information on the models is detailed in our recent study of RTS,S in settings with vectorial capacities oscillating seasonally around fixed values, including their main assumptions and the model fitting (Text S1) [24].

The 14 models were parameterized using 61 datasets and fit against 10 epidemiological outcome measures described previously [21], [24]. The contribution of each outcome was calibrated using a goodness of fit statistic reflected by the weighted sum of each log-likelihood contribution. The fit was confirmed if the models adequately reproduced the age-specific patterns of infection and morbidity from the original data sets.

Each model run was a stochastic simulation of a stable population of 100,000 people, run with five day time steps for an observation period of 10 years. The simulated populations had approximately stationary age-distribution typical of rural Tanzania, achieved by adjusting birth and out-migration rates to the required values [31].

The models were programmed in C++ as part of the open source software platform OpenMalaria (http://code.google.com/p/openmalaria/). SAS GPLOT was used to generate the figures (SAS Institute Inc., Cary, NC, USA, version 9.2 for Windows).

### Transmission settings

**Figure 1:**
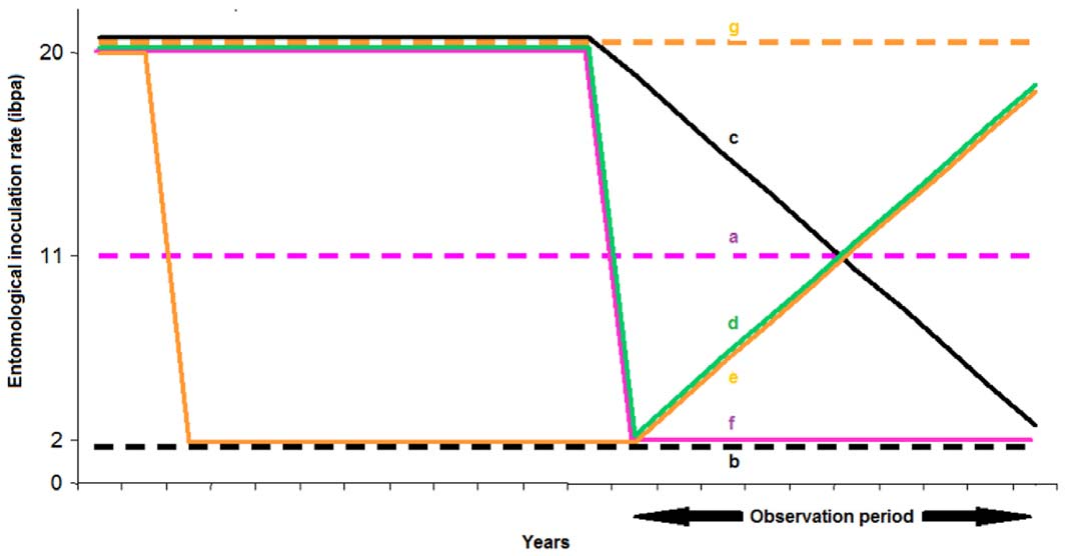
Simulated transmission settings. Legend: a (dashed purple line): EIR = 11 ibpa; b (dashed black line): EIR = 2 ibpa; c (solid black line): EIR decreasing from 20 down to 2 ibpa over 10 years; d (solid green line): EIR increasing after brief suppression from 2 to 20 ibpa over 10 years; e (solid orange line): EIR increasing from 2 to 20 ibpa after being suppressed for 10 years; f (solid purple line): EIR suppressed from 20 down to 2 ibpa, and subsequently maintained at 2 ibpa; g (dashed orange line): EIR = 20 ibpa.

In the absence of vaccination, baseline transmission was modeled to follow the seasonal patterns observed in Namawala, Tanzania, as in previous studies [22], [24], but with the EIR scaled to be in the range of 2 ibpa to 20 ibpa. Although transmission becomes less stable at the lower end of this range, a consistent cycle of transmission was assumed for the purposes of the modeling, unless changed as described below. In this range, comparable to EIRs achievable with existing interventions in most of rural Africa, the previous modeling studies suggest that RTS,S will be highly effective. In each case, the initial immune status of the simulated humans was set to that of a population recurrently exposed to an EIR of 20 ibpa. Simulations of vector control programs were then implemented so that in the absence of vaccination, the profile of transmission during the period of the vaccination program followed one of the following seven patterns (Figure 1):

- EIR = 11 ibpa, approximately, throughout the ten year observation period.
- EIR = 2 ibpa, approximately, throughout the ten year observation period.
- Decreasing: EIR decreasing linearly from 20 down to 2 ibpa over a ten year period.
- Increasing after brief suppression: EIR initially reduced to 2 ibpa, then increasing linearly to 20 ibpa over the ten year observation period.
- Increasing after ten years suppression: EIR which had been brought from 20 down to 2 ibpa and maintained there for ten years, increasing again up to 20 ibpa over the ten year observation period due to decreased use of IRS.
- Suppressed: EIR decreased rapidly from 20 down to 2 ibpa, and subsequently maintained at 2 ibpa during the ten year observation period.
- EIR = 20 ibpa, approximately, throughout the ten year observation period.

Settings a, b, and g correspond to settings with stable transmission. In these cases, the populations were simulated as having been exposed to these EIRs for a warm-up period corresponding to a full lifetime, before the start of the observation period during which vaccines were introduced.

Settings c, d, e, and f had changing baseline levels of transmission. The simulated populations were exposed to an annual EIR of 20 ibpa during the ‘warm-up’ period but then changes in the transmission, and hence the EIR during the observation period were induced by simulating IRS programs carried out in parallel with the simulated vaccination programs. The simulated coverage and effectiveness of the IRS programs were adjusted to achieve approximately linear trends in EIR in the absence of vaccination [35], [36].

### Vaccine efficacy and half life

RTS,S was modeled to have an efficacy of 60% against the force of infection from the time of completing the full, three dose vaccination course. The same moderate level of heterogeneity among individuals in the level of protection was assumed as in previous simulations [24], [37]. Efficacy after a single dose and two doses was modeled as 40% and 50%, respectively. The intention-to-treat analysis included in the most recent RTS,S trial publication, suggests that a substantial proportion of the efficacy may be reached before completion of the vaccination schedule [2]. A field study including a two-dose schedule using immunologic endpoints found that all subjects had significant responses, however, as there are no protective thresholds established, there is no way to determine the associated efficacy [38]. Immunologically it is reasonable to assume that three doses will strengthen the immune response and lengthen the duration of protection as compared to fewer doses. Previous analyses of our models showed that results are not very sensitive to the (extreme) assumption that the vaccine has no efficacy in those who do not receive all three doses. In the case of EPI, the results change very little given that coverage with all three doses tends to be very high. In case of school-based and mass campaigns, the study found that effectiveness against mortality relative to previous levels was 0.91 (95% CI 0.61–1.65) or 0.58 (95% CI 0.04–1.00) respectively [24]. Efficacy against clinical disease in these models is lower than efficacy against force of infection [24], [37], as is the efficacy against severe disease found in a clinical trial. Field studies have not yet associated efficacy against clinical disease with efficacy against deaths.

Vaccine efficacy against force of infection was modeled to decay exponentially with a half-life of ten years. The relatively short and/or small field studies undertaken to date have not provided clear trial data on decay of efficacy of RTS,S. Past modeling has suggested that vaccine impact is not particularly sensitive to the length of the half-life in the 4–10 year range [22], and has been used to derive calibration curves that can be used to convert the predictions for long half-life vaccines to approximate values for shorter half-lives (Text S1) [24]. Clinical efficacy observed in trials is expected to wane more rapidly than the underlying efficacy against the force of infection [24] because the age-pattern of disease in vaccinees begins to mimic that of an area with lower transmission in which disease is delayed to an older age [39], [40].

### Vaccine delivery strategy

Four delivery strategies were considered:

- EPI. Coverage of 89%, based upon Tanzania's DTP3 coverage in 2002. 95% of subjects received one dose, 93% two doses, and 89% all three doses at intervals as described in the introduction.
- EPI plus catch-up. In addition to delivery through EPI (i above), as the vaccine is first introduced into an area, children too old for EPI up to 18 months of age were vaccinated at monthly campaigns with three doses. No child received more than three doses. Coverage of children through campaigns was 80% selecting randomly for each dose, thus 51% of children up to 18 months of age received all three doses.
- EPI plus vaccination of school children. In addition to delivery through EPI (i above), primary school age children (aged 6 to 11 years) were vaccinated in monthly campaigns with three doses at program initiation only. Coverage of children through campaigns was 80% [41] selecting randomly for each dose, thus 51% of children 6–11 years of age received all three doses. Each subsequent year, only new students were vaccinated. Children who were initially vaccinated as infants received a single (booster) dose upon entry into the school system, also at 80% coverage.
- Mass vaccination. People from all ages were vaccinated in an initial campaign for three doses at one month intervals. Coverage through campaigns was 80% [42] selecting randomly for each dose, thus 51% of people of all ages receive all three doses. Subsequent campaigns delivered a single booster to 80% of the population every five years.

Table 1 summarizes the scenarios, and their justifications, used in the simulations.

**Table 1:** Summary of scenarios used in simulations.

Category	Assumption	Value	Source
**Transmission**	Unchanging	11 ibpa	Estimate
	Unchanging	2 ibpa	Estimate
	Decreasing	20 to 2 ibpa over 10 years	Estimate
	Increasing after brief suppression	2 to 20 ibpa over 10 years	Estimate
	Increasing after 10 year suppression	2 to 20 ibpa over 10 years	Estimate
	Suppressed	20 to 2 ibpa and maintained	Estimate
	Unchanging	20 ibpa	Estimate
**Vaccine coverage (1, 2 or 3 doses)**	EPI	95%; 93%; 89%	WHO, http://apps.who.int/immunization_monitoring/en/globalsummary/countryprofileselect.cfm; Accessed November 30, 2011
	Campaign or booster dose	80% at each dose, selecting randomly	[41], [42]
**Efficacy**	3 doses	60%	[24], [37]
	2 doses	50%	Estimate
	1 dose	40%	Estimate
**Half-life**	Exponential decay	10 years	Estimate, [22], [24]
**Delivery strategy**	EPI	Up to 3 doses at 6, 10, and 14 weeks of age	WHO, http://www.who.int/immunization/policy/Immunization_routine_table2.pdf; Accessed November 30, 2011
	EPI plus catch-up campaign	EPI plus up to 3 doses for children up to 18 months	Estimate
	EPI plus school campaign	EPI plus single booster at school entry or 3 doses for children 6 to 11 years	[41]
	Mass campaign	3 doses to people of all ages with single booster every 5 years	Estimate

**Legend**: EPI = Expanded Programme on Immunization; ibpa = infectious bites per annum.

## Results

### Number of doses required

**Figure 2:**
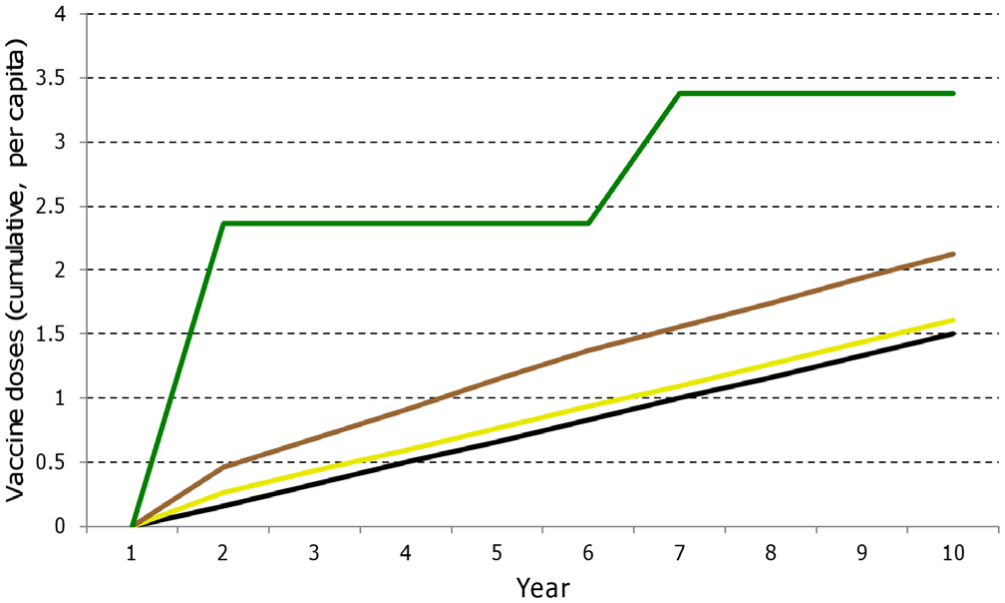
Cumulative doses per capita, by delivery strategy, in a dynamic population. Legend: Black = EPI; Yellow = EPI plus catch-up of children up to 18 months of age; Brown = EPI plus school-based immunization of children 6–11 years of age; Green = Mass campaigns in entire population. The per capita estimates assume a dynamic population, modeled upon the age distribution in rural Tanzania.

Figure 2 shows the cumulative number of doses per capita (in the all age population) required for each delivery strategy over a ten year period. EPI (strategy i), vaccinating infants from six weeks of age, required the fewest doses, ranging from 0.67/capita over five years to 1.50/capita over ten years. For a population of 100,000 this is equivalent to 67,000 doses over five years and 150,000 over ten years. An EPI plus catch-up strategy (ii), which involves a single campaign in the first year, targeting the cohort of children up to 18 months of age, required an additional 0.10 doses/capita. The EPI plus school-based strategy (iii), which assumed 51% of kids fully vaccinated, required 1.14 doses/capita over five years and 2.12/capita over ten years. The mass campaign (strategy iv), which assumed 51% of the population fully vaccinated, progressed in a stepped fashion. The year-one campaign required 2.37 doses/capita, or 237,000 for a population of 100,000. A total of 3.38 doses/capita was required after the booster in year five.

### Impact of delivery strategy on transmission

**Figure 3A–F:**
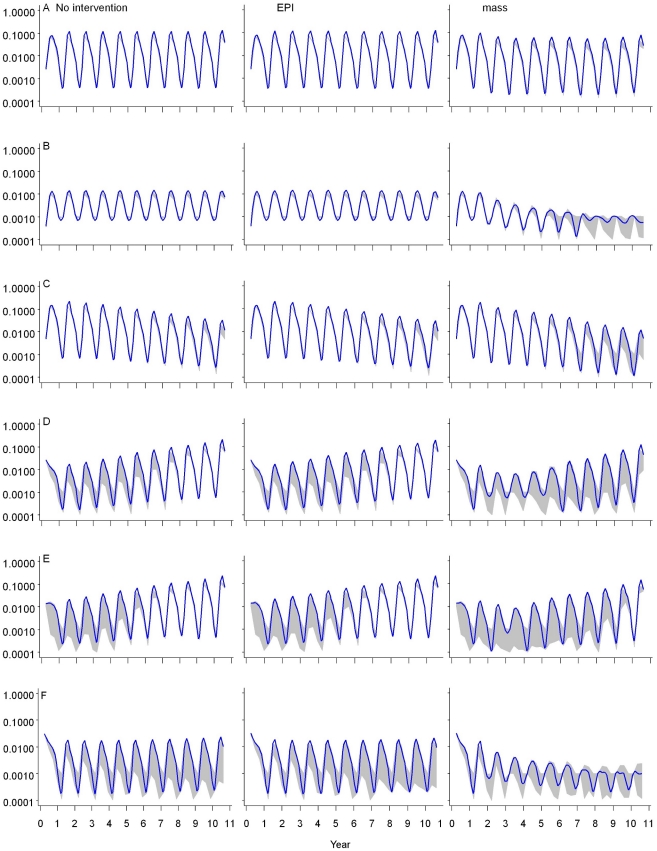
F. Modification of transmission trends, by delivery strategy, over ten years. Legend: The first column corresponds to no vaccine, the second corresponds to vaccine delivered through EPI and the third corresponds to vaccination through mass campaigns to all ages of the population. The vertical axis for each panel is EIR on a log scale measured in infectious bites per person per night. The horizontal axis for each panel is time in years. The blue line corresponds to the median transmission of the 14 models, while the grey shading corresponds to an envelope enclosing 95% of all simulations. Panel A: EIR = 11 ibpa; B: EIR = 2 ibpa; C: EIR decreasing from 20 down to 2 ibpa over 10 years; D: EIR increasing after brief suppression from 2 to 20 ibpa over 10 years; E: EIR increasing from 2 to 20 ibpa after being suppressed for 10 years; F: EIR suppressed from 20 down to 2 ibpa, and subsequently maintained at 2 ibpa.

Figure 3 presents the impact of RTS,S, delivered through EPI or mass vaccination, on malaria transmission over a ten year period, in each transmission setting. These were similar to those with EPI alone. The results for the setting of EIR = 20 ibpa is not shown as it was similar to the setting of EIR = 11 ibpa.

The annual patterns were a result of seasonality of vector abundance and the timing of the IRS rounds, if implemented. Consistent with past analyses using a fixed level of transmission [20], [23], RTS,S did generally not have much impact on transmission in the higher EIR ranges modeled. At EIR = 2 ibpa, whether naturally occurring, or suppressed to that level through use of IRS, the vaccine lowered transmission initially when delivered through mass campaigns but the effect leveled off after seven years.

### Impact on uncomplicated malaria cases

Table 2 presents the number of uncomplicated malaria cases experienced in each setting and the percentage of those cases which could be averted by RTS,S, depending on the delivery strategy.

**Table 2:** Uncomplicated cases averted with RTS,S by transmission setting and delivery strategy.

	Cases/Person-year (without vaccine)	Percentage of cases averted (Range)			
Transmission setting		EPI	Catch-up (plus EPI)	School-based (plus EPI)	Mass campaign
**a) EIR = 11**	1.63	6.0 (4.8–7.0)	7.1 (5.6–8.4)	12.0 (8.6–13.1)	24.5 (20.2–30.6)
**b) EIR = 2**	1.38	5.2 (4.1–6.0)	6.6 (5.4–7.3)	17.6 (15.0–21.7)	73.0 (70.4–76.5)
**c) Decreasing (EIR 20 to 2)**	1.35	7.1 (5.5–8.0)	8.6 (6.6–9.7)	14.0 (10.0–16.4)	30.0 (25.4–37.9)
**d) Increasing after brief suppression (EIR 2 to 20)**	1.96	5.1 (3.7–6.8)	5.2 (3.7–7.0)	11.9 (7.8–20.5)	32.1 (26.4–59.7)
**e) Increasing after 10 year suppression (EIR 2 to 20)**	2.97	3.4 (2.5–4.5)	4.1 (3.0–5.8)	8.3 (5.2–12.6)	20.7 (16.7–50.2)
**f) Suppressed (EIR = 2)**	1.46	5.1 (3.0–6.1)	6.6 (4.6–7.8)	15.3 (9.9–17.8)	48.5 (20.2–50.1)
**g) EIR = 20**	1.53	6.0 (4.1–7.1)	7.0 (4.4–8.2)	10.0 (5.4–11.6)	15.5 (6.6–20.7)

**Legend**: The first column presents transmission settings. The second column presents the total estimated cases in the all-ages population of 100,000 people per person-year in the absence of vaccination. The estimate is the average over a 10 year period. The remaining columns present the percentage of cases averted by each delivery strategy, as the median and (range) estimated from 14 models.

All-age rates of uncomplicated disease in simulated stable settings were rather insensitive to the average level of EIR, corresponding to the data used to fit the models [32], [43], where the effect of varying transmission is to shift the age-pattern, rather than the overall incidence of uncomplicated disease. However, changes in EIR, and in particular an increase in transmission has a substantial effect on simulated incidence. In the absence of vaccination, each person was estimated to have between 1.35 cases per year (in a decreasing transmission setting) and 2.97 cases (setting where control was lost).

EPI averted 3.4–6.0% of malaria cases depending on the transmission, while EPI plus catch-up averted 4.1–8.6% of cases. EPI plus school-based strategies averted 8.3–17.6% of cases, depending on transmission, while mass campaigns averted 15.5–73.0% of cases. The range of estimates was widest for the mass campaign strategies and most narrow for EPI strategies, and overlapped for these two strategies in transmission scenario g (EIR = 20 ibpa).

**Figure 4A–F:**
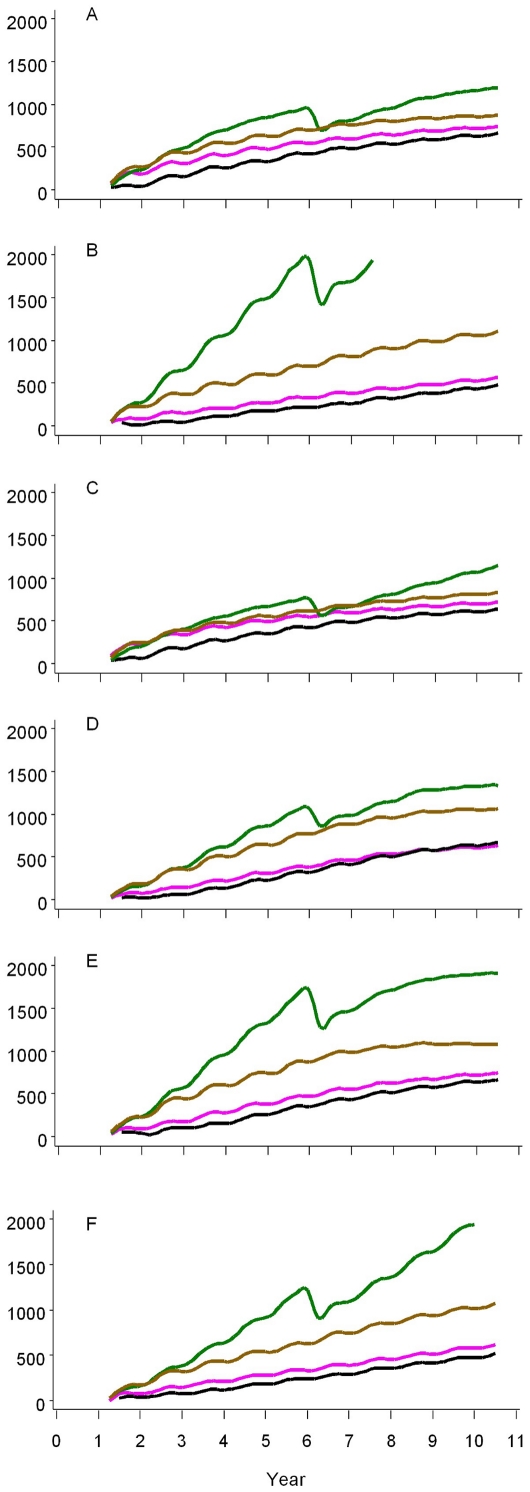
F. Uncomplicated cases averted per 1000 doses administered, by delivery strategy and transmission setting, over ten years. Legend: Vertical axis for each panel is number of cumulative uncomplicated cases averted per 1000 doses administered. Horizontal axis is years. Vaccination is assumed to begin at year one with the vaccination schedule completed three months later for the first subjects. Black = EPI; Purple = EPI plus catch-up of children up to 18 months of age; Brown = EPI plus school-based immunization of children 6–11 years of age; Green = Mass campaigns in entire population. Panel A: EIR = 11 ibpa; B: EIR = 2 ibpa; C: EIR decreasing from 20 down to 2 ibpa over 10 years; D: EIR increasing after brief suppression from 2 to 20 ibpa over 10 years; E: EIR increasing from 2 to 20 ibpa after being suppressed for 10 years; F: EIR suppressed from 20 down to 2 ibpa, and subsequently maintained at 2 ibpa.

Figure 4 presents the ratio of uncomplicated cases averted to1000 doses administered. This standardized measure allows comparison of the efficiency of different delivery strategies across transmission settings.

Across all settings, the cumulative number of cases averted per1000 doses was highest with mass campaigns, followed by EPI plus school, EPI plus catch-up, and EPI alone. After ten years, EPI and EPI plus catch-up averted 500–700 cases per 1000 doses across all settings. An EPI plus school-based strategy averted 800–1100 cases per 1000 doses across all settings. Results from mass campaigns were more variable. In settings with EIR = 11 ibpa (Panel A), decreasing EIR (Panel C), and increasing EIR after a brief suppression (Panel D), RTS,S averted 1100–1300 cases per 1000 doses. In a setting with EIR increasing after ten years suppression (Panel E), it averted approximately 2000 cases per 1000 doses from year eight onwards but the effect appeared to be plateauing. In a setting with EIR suppressed to 2 ibpa (Panel F), it averted a similar ratio of cases over the ten year period, but the curve suggested increasing efficiency beyond year ten. The highest efficiency was seen in EIR = 2 ibpa where the models predicted that RTS,S could avert more than 2000 cases per 1000 doses after 5–8 years.

### Impact on malaria deaths

**Figure 5A–F:**
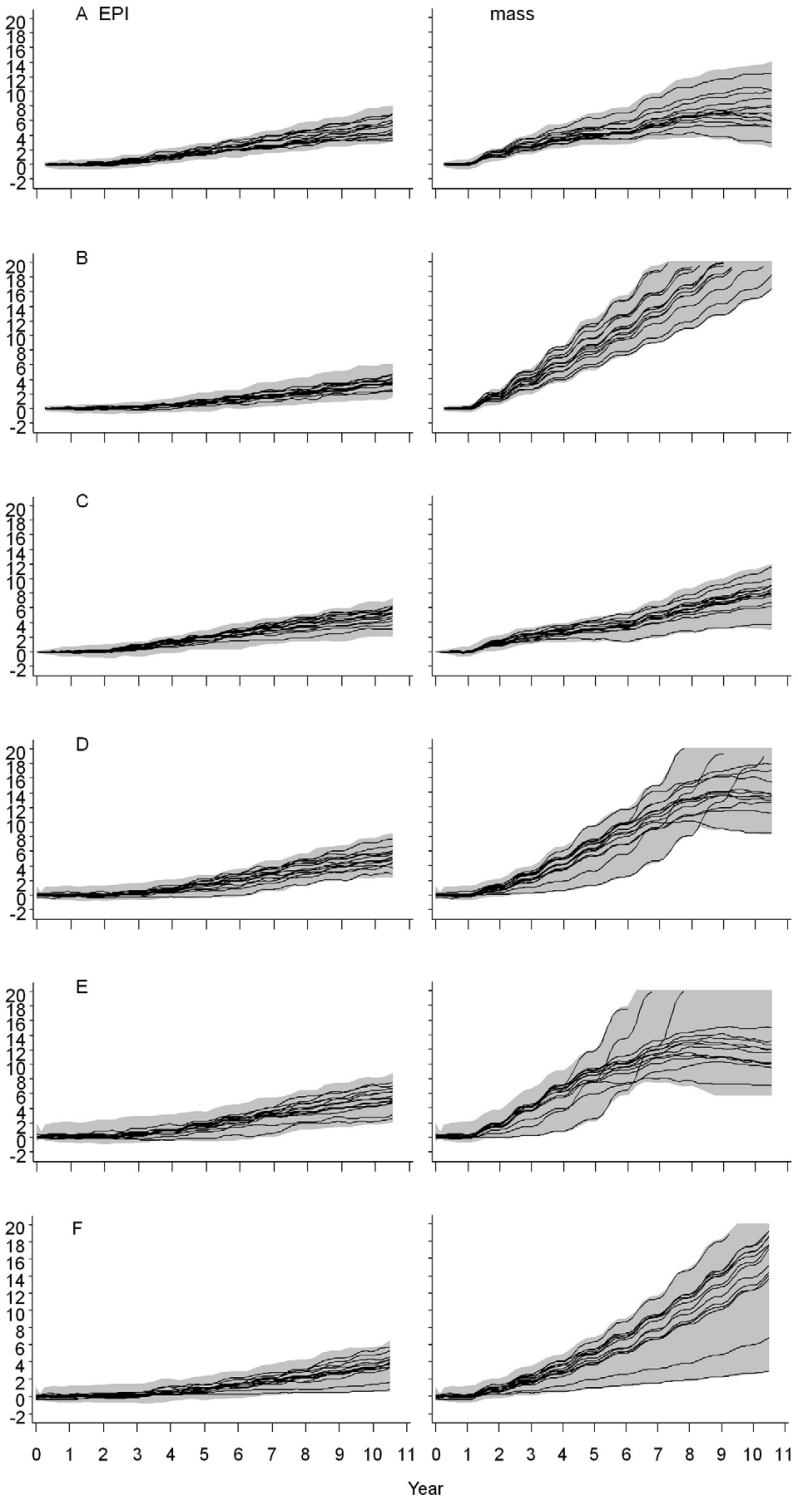
F. Deaths averted per 1000 population, by transmission setting, over ten years. Legend: The first column corresponds to vaccine delivered through EPI and the second corresponds to vaccination through mass campaigns to all ages of the population. The vertical axis for each panel is number of cumulative deaths averted per 1000 population. The horizontal axis for each panel is years. The black lines correspond to the median effect estimated by each of the 14 models, while the grey shading corresponds to an envelope enclosing 95% of all simulations. Vaccination is assumed to begin at year one with the vaccination schedule completed three months later for the first subjects. Panel A: EIR = 11 ibpa; B: EIR = 2 ibpa; C: EIR decreasing from 20 down to 2 ibpa over 10 years; D: EIR increasing after brief suppression from 2 to 20 ibpa over 10 years; E: EIR increasing from 2 to 20 ibpa after being suppressed for 10 years; F: EIR suppressed from 20 down to 2 ibpa, and subsequently maintained at 2 ibpa.

Table 3 presents the number of malaria deaths, per 1000 person-years, occurring in each transmission setting, and the percentage of deaths which simulations suggested could be averted by RTS,S. Figure 5 complements this data, presenting the cumulative deaths averted per 1000 persons over 10 years for each of the 14 models.

**Table 3:** Deaths averted with RTS,S by transmission setting and delivery strategy.

	Deaths/1000 person-years (without vaccine)	Percentage of deaths averted (Range)			
Transmission setting		EPI	Catch-up (plus EPI)	School-based (plus EPI)	Mass campaign
**a) EIR = 11**	3.8	13.7 (10.3–16.2)	14.8 (11.9–16.7)	15.9 (10.3–20.9)	18.8 (7.1–25.7)
**b) EIR = 2**	3.7	10.2 (7.2–14.3)	11.9 (9.0–16.7)	22.4 (14.5–26.0)	68.1 (65.5–72.2)
**c) Decreasing (EIR 20 to 2)**	3.3	15.5 (11.3–17.7)	17.2 (14.1–20.9)	19.0 (12.4–20.7)	25.2 (13.1–32.8)
**d) Increasing after brief suppression (EIR 2 to 20)**	5.0	10.3 (8.0–11.5)	9.8 (7.3–11.4)	14.9 (9.0–20.1)	28.3 (13.8–58.5)
**e) Increasing after 10 year suppression (EIR 2 to 20)**	7.8	6.8 (3.1–8.3)	7.2 (4.9–9.0)	9.4 (3.7–12.1)	14.2 (1.8–42.5)
**f) Suppressed (EIR = 2)**	3.7	10.5 (5.6–12.7)	11.7 (4.4–14.7)	18.7 (10.8–21.1)	45.4 (21.0–47.5)
**g) EIR = 20**	3.8	13.3 (9.6–15.7)	14.0 (9.7–17.8)	13.6 (8.1–18.8)	9.9 (1.6–15.7)

Legend: The first column presents transmission settings. The second column presents the total estimated malaria deaths in the all-ages population of 100,000 people per person-year in the absence of vaccination. The estimate is the average over a 10 year period. The remaining columns present the percentage of deaths averted by each delivery strategy, as the median and (range) estimated from 14 models.

Delivery through EPI was estimated to avert 6.8–15.5% of malaria deaths, depending on the transmission, while EPI plus catch-up averted 7.2–17.2% of deaths. EPI plus school-based strategies averted 13.6–22.4% of malaria-related deaths, depending on transmission, while mass campaigns averted 9.9–68.1% of these deaths. The upper estimates of the ranges for mass campaigns likely reflected the effect of herd immunity magnifying the impact of the vaccine. The ranges of model medians from EPI and mass campaign strategies overlapped in all settings except b and f where EIR was at or suppressed to 2 ibpa, while in setting g (EIR = 20 ibpa), the point estimate of deaths averted was higher for EPI than from mass campaigns. The ranges of estimates were widest for the mass campaign strategies and narrowest for EPI strategies. This is illustrated by the variation between model medians shown in Figure 5. The only setting where all model medians showed higher impact from mass campaigns than from EPI was in settings with EIR = 2 ibpa (Panel B). A high level of uncertainty was associated particularly with settings where the EIR was increasing after a brief suppression (Panel D), increasing after a ten year suppression (Panel E), or continuously suppressed to 2 ibpa (Panel F). The ranking of the different models in mortality benefits differed by vaccination strategy and transmission intensity, making it challenging to identify which elements of model structure drove this uncertainty.

**Figure 6A–F:**
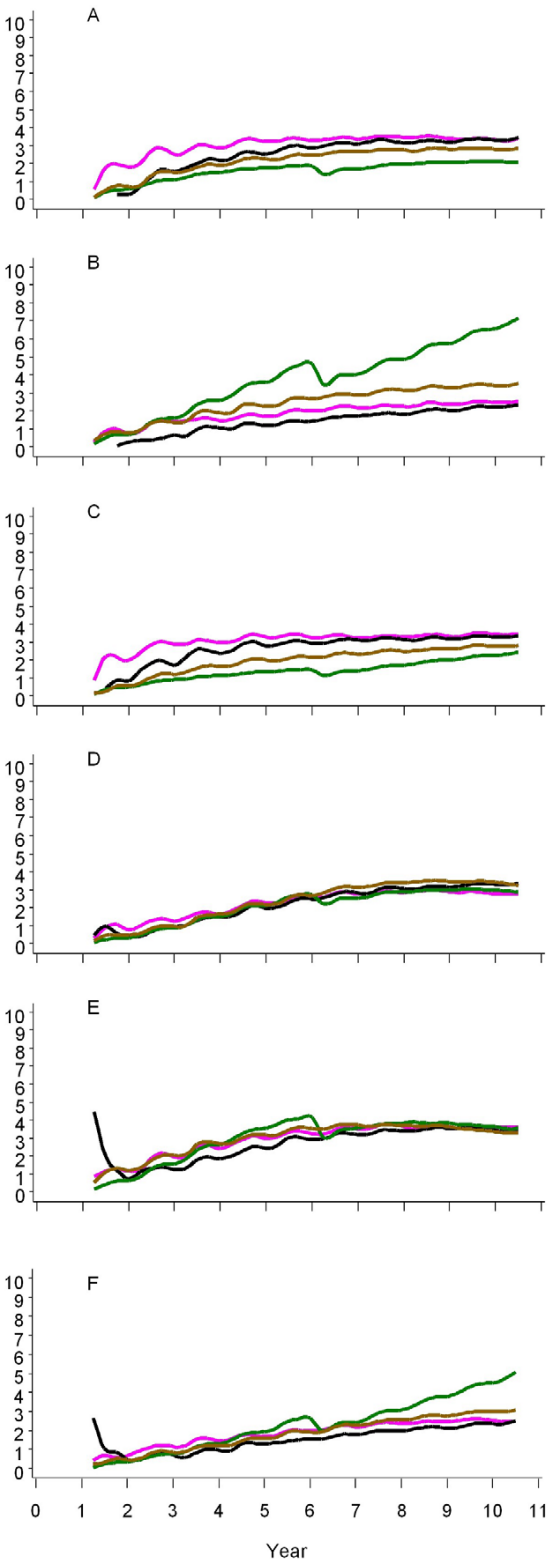
F. Deaths averted per 1000 doses administered, by delivery strategy and transmission setting, over ten years. Legend: Vertical axis for each panel is number of cumulative deaths averted per 1000 doses administered. Horizontal axis is years. Vaccination is assumed to begin at year one with the vaccination schedule completed three months later for the first subjects. Black = EPI; Purple = EPI plus catch-up of children up to 18 months of age; Brown = EPI plus school-based immunization of children 6–11 years of age; Green = Mass campaigns in entire population. Panel A: EIR = 11 ibpa; B: EIR = 2 ibpa; C: EIR decreasing from 20 down to 2 ibpa over 10 years; D: EIR increasing after brief suppression from 2 to 20 ibpa over 10 years; E: EIR increasing from 2 to 20 ibpa after being suppressed for 10 years; F: EIR suppressed from 20 down to 2 ibpa, and subsequently maintained at 2 ibpa.

The efficiency of RTS,S averting deaths is presented as a ratio of the number of deaths averted to1000 doses administered (Figure 6). The results for the setting of EIR = 20 ibpa is not shown as it was similar to the setting of EIR = 11 ibpa.

Across all settings, the cumulative number of deaths averted per 1000 doses of RTS,S generally leveled off at a maximum of 3–4. Such efficiency was realized only after approximately seven years in settings with increasing transmission, either after brief or ten year suppression (Panels D and E), while it was reached after only two to three years in the setting with decreasing transmission (Panel C) with delivery through EPI plus catch-up or EPI alone. The highest impact was approximately 5 (Panel F) to 7 (Panel B) deaths averted per 1000 doses ten years after beginning mass campaigns in settings where the EIR was naturally at or suppressed to 2 ibpa. In these settings, RTS,S not only protected those vaccinated, but also reduced transmission (Figure 3), likely leading to herd immunity and rapidly increasing impact. As reflected by the ranges in Table 3, these estimates are most precise for EPI and least precise for mass campaigns. All of these estimates depend on the assumption of a persistent effect of vaccination, but can be adjusted to allow for shorter durations using published calibration data [24].

## Discussion

Modeled estimates of the effect of the RTS,S vaccine allow for comparisons of uncomplicated cases and deaths averted, and the efficiency of different delivery strategies to avert those cases and deaths, in a variety of malaria transmission settings.

In most transmission settings studied, an approximately proportional relationship between the number of deaths averted and the number of doses administered through EPI, EPI plus catch-up and EPI plus school-based campaigns, was observed over the ten year period simulated. That is, among the delivery strategies that target a portion of the population, the greater the number of doses administered, the greater the total number of lives that can be saved.

The EPI strategy immunized the fewest people over ten years, and consequently averted the fewest deaths, but with a relatively high efficiency per 1000 doses administered across transmission settings. This strategy also provided the most consistent impact across transmission settings studied. EPI has demonstrated its feasibility and is compatible with current clinical trial and regulatory plans. The opportunity to make use of the thus far successful EPI infrastructure is a strong argument to use this strategy in (all) transmission settings where it is likely to be cost-effective to deliver RTS,S this way.

Supplementing EPI delivery with a one-time catch-up vaccination reaching children below 18 months may be particularly appealing in an environment with decreasing transmission (setting c). The catch-up delivery round would require additional investment and planning beyond that seen for EPI, although most likely less than required for repeated school-based or mass campaigns.

School-based delivery strategies did not increase efficiency in any of the transmission settings studied, so its main benefits are in reaching a greater number of people. A recent pilot of school-based strategies for delivering human papilloma-virus (HPV) vaccine could be part of the momentum to strengthen and expand school-based strategies over time [44].

The findings for mass campaigns were more complex. Mass campaigns reached many times more people than EPI alone, and simulations suggested this delivery strategy could be expected to avert more cases and deaths in some situations. Highest efficiency was seen in very low transmission settings (e.g. EIR of 2 ibpa), whether naturally occurring or achieved by other interventions (settings b and f). Efficiency decreased as transmission increased, making mass campaigns much less efficient than EPI at EIRs above 2 ibpa.

The impact of mass campaigns varied the most between different transmission settings, and there were wide ranges in the model predictions. In contrast to EPI, the expected benefits of mass vaccination were highly sensitive to model assumptions about transmission heterogeneity and immunity. The amount of field data on older children in semi-immune populations and adults is small, and there is a clear need to improve the evidence base for parameterizing these aspects of the models. Impact estimates from mass vaccination in low transmission settings were magnified by herd immunity. Vaccinating a large proportion of the population could decrease the likelihood that mosquitoes bite a person with circulating parasites, thereby decreasing the number of infectious mosquitoes in a region. Herd immunity is unlikely to arise with EPI strategies given that the vaccinated infants represent a relatively small portion of the population so would not significantly impact the number of circulating mosquitoes with parasites.

Part of the uncertainty about impact of mass-vaccination (also reaching adults) arose because relatively sparse data about disease incidence in older people were available when the models were fitted [32], [33]. In our models, the main effect of reducing transmission on disease is to shift morbidity to older ages. It is not clear how important this shift is in settings where transmission is currently reducing. If this shift is less important, independently of the effects of the vaccine, we might have expected to see larger effects of reducing transmission on morbidity and mortality rates. In contrast to most field datasets, the presented rates are calculated over the entire population and simulated events in adults dilute the effects on children. Thus, caution is advised when interpreting model results of mass campaigns until further data is available.

Mass vaccination also appears the most distant potential RTS,S delivery strategy. New clinical trials, in older age groups potentially with different safety questions and needing different doses, may be necessary. Regulatory, cost-effectiveness and feasibility considerations related to mass campaigns of a multi-dose vaccine given at one-month intervals, may also need to be addressed. The findings suggest that further work is needed to more clearly understand the impact and feasibility of mass campaigns, potentially when partnered with other interventions to rapidly decrease transmission. The potential for significant impact in some settings may make it worth beginning to anticipate such work.

Estimates of efficiency in this paper are expressed using the ratio of deaths averted per 1000 doses of vaccine. This can be converted to deaths averted per 1000 vaccinees, a ratio used by the GAVI Alliance (GAVI) when considering the impact of various vaccines it supports financially [45]. The ratio of deaths averted by pneumococcal conjugate vaccine in African infants is estimated from trial data to be 7 per 1000 vaccinees (59 deaths averted in 8000 vaccinees) [46]. Immunizing 1000 infants with RTS,S via EPI, given the coverage assumptions stated earlier, equates to delivering approximately 2770 doses of vaccine. Given the ratios under transmission settings above, simulations suggest RTS,S in EPI would avert 2–4 deaths per 1000 doses, equivalent to 6–11 deaths per 1000 vaccinees. While one should be cautious comparing data from field studies with those from modeling studies, these ratios suggest a favorable comparison with the pneumococcal vaccine, for which implementation planning is progressing rapidly in Africa and some $3 billion in financing have been committed [47].

The findings across the different settings suggest that for understanding the potential impact of pre erythrocytic vaccines, it is probably sufficient to use fixed transmission settings in modeling efforts, even for situations where the underlying baseline transmission is believed to be decreasing or increasing. The simulations used in this paper used IRS to create trends in transmission. Effects would have been slower if the transmission changes were induced by LLIN use, but there is no reason to assume that the relative impact of the delivery strategies would be different.

The effect for RTS,S was expected to be at maximum in the transmission range studied in these simulations (2–20 ibpa). It is reasonable to expect that EPI, EPI plus catch-up and EPI plus school-based strategies will have comparable effects to those seen in this paper at somewhat higher transmission settings than studied. Although cost-effectiveness estimates for EPI are available [26], the other impact estimates could be further informed by cost and cost-effectiveness analyses which were beyond the scope of this paper.

### Conclusion

The development of the RTS,S malaria vaccine is progressing. If the vaccine is shown to be safe and efficacious in the current phase III trial, as has been shown in earlier trials, and it is made available to countries in Africa, there will be a complex decision-making process to determine how and where it should be used. In order to help this process, the authors studied the potential impact and efficiency of RTS,S delivered through various strategies and under changing levels of malaria transmission patterns, although findings suggest that it is probably sufficient for future simulations to consider constant EIR levels. The type of impact sought, whether on mortality and morbidity, or on transmission as well, together with assumptions about feasibility of different delivery strategies, may lead to different conclusions about the role of RTS,S.

Findings support further investigation of RTS,S to determine its potential contribution to malaria transmission reduction through high coverage of an all age population in low transmission settings. At 2 ibpa, mass campaigns could avert the most deaths and decrease transmission; however, a lot of uncertainty was associated with these findings. Also, the feasibility of mass campaigns delivering a multi-dose immunization requires assessment. Further consideration of the regulatory implications, clinical studies required to address questions of the extent of the benefit, and other product development activities will be important to anticipate, prior to RTS,S being used in mass campaigns. Such considerations would benefit from early planning due to their lengthy nature.

The findings also support going forward with EPI delivery as an initial strategy for RTS,S. If delivered via established EPI systems, RTS,S could avert approximately 6–11 deaths per 1000 vaccinees in all examined settings, an efficiency consistent with or superior to other new vaccines being implemented across Africa. However, it would have little effect on transmission when delivered via EPI. The number of events averted increases somewhat with the EPI plus catch-up and the EPI plus school-based strategies. The increase is essentially proportional to the larger population covered, and because that larger population coverage is modest, it is not an enormous added benefit. These results, combined with those of other modeling studies, should provide information to help policy-makers at international and country-levels to take evidence-based decisions on optimal strategies to avert malaria cases and deaths in Africa.

## Supporting Information

Text S1 **Ensemble Modeling of the Likely Public Health Impact of a Pre-Erythrocytic Malaria Vaccine.**
(PDF)
